# Short-term effect of orthokeratology lens wear on choroidal blood flow in children with low and moderate myopia

**DOI:** 10.1038/s41598-022-21594-6

**Published:** 2022-10-21

**Authors:** Qing Zhu, Qi Zhao

**Affiliations:** Department of Ophthalmology, The Second Hospital of Dalian Medical University, Dalian, 116027 China

**Keywords:** Medical research, Paediatric research, Clinical trial design

## Abstract

We investigated changes in choroidal vascularity and choriocapillaris blood perfusion during orthokeratology (Ortho-K) lens wear. Sixty-two children with low to moderate myopia were enrolled. The Ortho-K group (n = 42) continuously wore Ortho-K lenses for 3 months, and the controls (n = 20) wore single-vision distance spectacles. All of the patients were instructed to return for follow-up visits after 1 day, 1 week and 1 month and 3 months of treatment. The subfoveal choroidal thickness (SFChT), choroidal vascularity [including the total choroidal area, luminal area, stromal area, and choroidal vascularity index (CVI)] and percentage of choriocapillaris flow voids (FV%) were determined with a Cirrus HD-OCT instrument. Additionally, ocular parameters were measured. In the Ortho-K group, the SFChT significantly increased by 12.61 ± 5.90 μm, the CVI was significantly increased by 2.99 ± 2.07% and 3.01 ± 2.32% on the horizontal and vertical scans respectively, and the FV% was significantly decreased by 0.89 ± 0.34% from baseline at the 1-week visit (all p < 0.001). The choroidal parameters remained unchanged at the 1-month and 3-month follow-ups with respect to the 1-week follow-up. In the control group, the choroidal parameters did not change significantly at 1 month (all p > 0.05). At the 3-month visit, the changes in the axial length (AL) and vitreous chamber depth (VCD) were significantly greater in the control group than in the Ortho-K group (0.14 ± 0.23 and 0.03 ± 0.05 mm in AL, 0.15 ± 0.23 and 0.06 ± 0.03 mm in VCD respectively). Our longitudinal study showed several choroidal parameter changes in the early stage in Ortho-K lens wearers with low to moderate myopia, and these changes persisted over 3 months. We speculate that Ortho-K lenses regulate choroidal thickness and blood perfusion, affecting myopia development.

## Introduction

Myopia is one of the most common eye diseases worldwide, with a prevalence of 80–90% in young adults in some parts of East and Southeast Asia^1–3^. Recent meta-analyses have suggested that close to half of the world’s population could be myopic by 2050, with high myopia in up to 10% of the population^4^. Myopia is characterized by excessive axial elongation. High myopia is associated with an increased risk of developing a series of complications, such as degenerative retinopathy, lacquer cracks, choroidal neovascularization, and posterior staphylomas^5–7^. Therefore, the prevention of myopia, particularly high myopia, has become an important international public health priority.

Many clinical studies have reported decreases in choroidal thickness (ChT) in myopic humans^8–12^ and other animals (such as chicks^13,14^ and guinea pigs^15,16^) with negative lens-induced myopia (LIM) and form deprivation myopia (FDM), the extent of which are significantly correlated with the severity of myopia^13,17,18^. The choroid has been hypothesized to play an intermediary role in the eye-growth signalling pathway from the retina to the sclera, with changes in ChT either reflecting or causing changes in eye growth^19^.

Orthokeratology (Ortho-K), one of the most effective and popular treatments for myopia control^20–22^, involves the use of overnight reverse-geometry rigid gas permeable contact lenses to flatten the central cornea and steepen the mid-peripheral cornea, thereby inducing peripheral myopic defocus^23^. Recently, studies have shown that patients treated with Ortho-K lenses experience choroidal thickening, especially in the large choroidal vascular layer^24,25^. Changes in ChT are caused by retinal defocus, and positive lens-guided myopia defocus causes an increase in ChT^26,27^ The mechanism of the choroidal thickening is believed to be primarily involved in the peripheral myopic defocus caused by Ortho-K lens wear, which is also one of the mechanisms controlling myopia growth. It is therefore speculated that the myopia control effects of Ortho-K involve the choroid. Considering the highly vascularized nature of the choroid, its vascularity could provide additional information about its morphology and physiology. However, ChT is not a comprehensive indicator to assess the status of choroidal vessels. The choroidal vascularity index (CVI) in structural optical coherence tomography (OCT) images and choriocapillaris flow voids (FVs) in optical coherence tomography angiography (OCTA) images can better delineate the global appearance of choroidal circulation and have shown good repeatability and reproducibility in studies by others^28–30^. Currently, there is no research on the changes in choroidal blood perfusion in patients treated with Ortho-K lenses. In this study, high-definition optical coherence tomography (HD-OCT) (CIRRUS HD-OCT 5000, SINGAPORE) was used to comprehensively analyze the short-term changes in choroidal vascularity and choriocapillaris blood perfusion in patients with low-to-moderate myopia treated with Ortho-K lenses.

## Methods

### Study design

This was a prospective, observational study. Patients were selected from among children diagnosed with myopia and treated with Ortho-K or single-vision spectacles in the Pediatric Ophthalmology Clinic, the Second Affiliated Hospital of Dalian Medical University. Our research followed the tenets of the Declaration of Helsinki, and we obtained informed consent from the patients’ caregivers and assent from the children before enrolment. Additionally, the study was approved by the Ethics Committee of the Second Affiliated Hospital of Dalian Medical University.

The inclusion criteria were as follows: age 8–12 years old at the time of enrolment; spherical equivalent refraction (SER = sphere + ½ cylinder) between −0.75 D and −6.00 D and best correct visual acuity of at least 0.00logMAR; and astigmatism in either eye ≤ 1.00 DC. Children with any known ocular pathology, history of ocular surgery, smoking history, systemic diseases, intraocular pressure (IOP) > 21 mm Hg, difference between the eyes > 8 mmHg, or a history of myopia control (either pharmacological or optical control) were excluded. The subjects were asked to avoid caffeine and alcohol intake during the 24 h before choroidal imaging. One eye (the right eye) was selected from each child for analysis.

Ophthalmic screening examinations, including noncycloplegic subjective refraction, binocular vision testing, ocular health evaluation, and IOP measurement, were conducted prior to formal enrolment. All of the patients were newly diagnosed with myopia by cycloplegic manifest refractions in our hospital before enrolment. The noncycloplegic refractive error was measured during this study since it was the most natural condition among the subjects in the study. The subjects in the Ortho-K group were fitted with a PARAGON CRT 100 Lens (Paragon Vision Sciences, Mesa, AZ, USA) for each eye. Both the children and their parents were taught lens handling and disinfection procedures. All of the subjects were required to wear their lenses on a daily basis during overnight sleep (for at least 7 consecutive hours). All of the patients were instructed to return for a follow-up visit after 1 day, 1 week, 1 month and 3 months. At each visit, the participants underwent a slit-lamp examination to check for contact lens-related complications and any adverse events. For the control group, the single-vision distance spectacle prescriptions were updated in our hospital before enrolment. All of the subjects were instructed to wear glasses during the day. In both groups, ocular biometric parameters were measured at baseline, 1 month, and 3 months, and choroidal parameters were measured at baseline,1 day, 1 week, 1 month, and 3 months.

### Experimental design

Measures for all of the subjects were completed between 2 and 5 pm on the relevant days to minimize any potential impact of diurnal variation^31^. At each visit, the patient's refractive status and visual acuity were first measured. If the patient demonstrated residual refractive error while wearing the Ortho-K lenses, additional refractive correction was performed during the video viewing (described below). Patients in the control group had their spectacle lenses adjusted according to their changes in refractive status. The participants then viewed a video at 5 m for a 20-min stabilization period with full distance correction for both eyes to reduce the influence of any previous visual stimuli (such as high accommodation^32^ or defocus^33^) on ChT. Choroidal images were obtained immediately after this period with a Cirrus HD-OCT device, as detailed below. Ocular biometric parameters, including the axial length (AL), central corneal thickness (CCT), aqueous depth (AD), and lens thickness (LT), were measured using the non-contact Biometer (lenstar LS 900, HAAG-STREIT AG, KOENIZ, SWITZERLAND). The vitreous chamber depth (VCD) was calculated as AL–(CCT + ACD + LT).

The Zeiss Cirrus HD-OCT Model 5000 instrument with Angioplex uses a so-called OCT-microangiography complex algorithm (OMAG) with a central wavelength of approximately 840 nm and an A-scan rate of 68 kHz. The OMAG identifies changes in the phase and intensity information of the OCT scans to quantify motion contrast^34^. The A scan depth was 2 mm, with an axial resolution of 5 μm and a transverse resolution of 15 μm. For eye tracking, Fast-Trac technology was implemented, and the retina was sampled at 15 frames per second to minimize motion artefacts. Only areas that might have been affected by motion artefacts were rescanned, decreasing the acquisition time. Structural OCT of the macular region was performed with high-definition 1-line raster scanning (HD 1 line 100×) centered on the fovea. One hundred B scans (each consisting of 1024 A scans) were obtained on each scan line to generate a single high-resolution scan with a length of 3 mm and a depth of 2.0 mm. Cirrus HD-OCT selective pixel profiling (SPP) provides better than average superimposed noise reduction imaging and can better display choroidal structures. Only the vertical and horizontal scans were used to analyse the subfoveal choroidal thickness (SFChT) and choroidal vascularity. The SFChT was manually measured as the distance between Bruch’s membrane (lower boundary of the retinal pigment epithelium [RPE]) and the choroid-scleral interface. The OCT images were analyzed with Image J version v2.0.0 (National Institutes of Health, Bethesda, MD, USA; available at imagej.nih.gov/ij/). (Supplementary Fig. S1) Due to the segmental nature of the choroidal blood supply^35^, the subchoroidal area with a width of 1.5 mm centred on the fovea was chosen as the region of interest (ROI). The specific analysis method was described in previous research^36^. First, the choroidal area was set with ImageJ ROI Manager. Then, three choroidal vessels with lumens larger than 100 μm were randomly selected by the Oval Selection Tool on the ImageJ tool bar, and the average reflectivity of these areas was determined. The average brightness was set at the minimum value to minimize the noise in the OCT image, and the image was then converted to 8 bits and adjusted with the Niblack Auto Local Threshold. The binarized image was converted to a red/green/blue (RGB) image again, and the luminal area was determined using the Threshold Tool. After adding the data of the distance on each pixel, the stromal area (SA), luminal area (LA), and total choroidal area (TCA) were automatically calculated, and the choroidal vascularity index was calculated as the LA/TCA. The light pixels were defined as the SA, and the dark pixels were defined as the LA.

For angiography, a 3 × 3 mm pattern with a resolution of 245 × 245 was chosen, with a mean distance of 12.2 microns between each scan, and each B scan was repeated 4 times at the same position. The choriocapillaris slab was defined by a layer starting at the outer boundary of the RPE–Bruch’s membrane and ending at approximately 20 μm beneath the RPE–Bruch’s membrane. A maximum projection was applied on the segmented volumes to generate the en face angiograms, and projection artefacts from the retinal vessels were removed. Choriocapillaris flow voids were defined as regions having no flow signals that were detectable by the threshold binarization algorithm, as previously described^29,30^. The en face OCTA choriocapillaris image was analyzed with ImageJ. (Supplementary Fig. S2) Because of poor image resolution at the scan edges, only the 2.5-mm-diameter circular region centered on the fovea was used for analysis. Automatic local thresholding was performed with the Phansalkar method using a radius of 15 pixels. The Phansalkar method selects darker regions of potentially low-contrast images to obviate small regional variations in image brightness^30^. The thresholded images were then analysed with the ‘Analyse Particles’ command, which measures and counts all thresholded areas greater than or equal to 20 pixels^2^ where there is a lack of flow information. All of the scans were reviewed to ensure correct segmentation and sufficient image quality. Any image with a double vessel pattern, motion artefacts, and/or segmentation errors extending more than three lines was excluded. To assess and affirm agreement within and between examiners, 30 images were selected and measured twice by two examiners (QZ and RL). The intra- and interrater reliability for image binarization were measured by the intra-class correlation coefficient (ICC) for absolute agreement. We also performed Bland–Altman plot analyses to determine the mean difference between the measurements. The Bland–Altman plots were constructed using MedCalc version 20.03 (Medcalc Software, Ostend, Belgium). After good agreement was verified, all of the scans were measured by the examiner QZ. The examiner was masked to the treatment of patients and the study visit at which the measurements were collected.

### Statistics

SPSS statistical analysis software, version 26.0 (IBM Corp., Armonk, NY, USA), was used for data analysis. The means and standard deviations of all continuous variables are presented unless otherwise stated. The normality of the data was assessed using the Shapiro–Wilk tests. The comparisons of baseline age, SE, AL, VCD and SFChT and other choroidal parameters in each study group were performed using the independent sample t test. Changes in the SFChT, choroidal parameters and other biometric parameters from baseline to each follow-up visit were examined using repeated-measurea analysis of variance (ANOVA), and Greenhouse–Geisser correction was applied to the degrees of freedom when the sphericity assumption was violated. Bonferroni’s adjustment for multiple comparisons was applied to all post hoc pairwise comparisons. A general linear model was used to compare the differences in ocular biometric parameters and choroidal parameters between groups at different follow-up time points. Potential confounding factors, including the baseline AL, VCD, SE, SFChT, and CVI on horizontal and vertical scans and changes in the CVI, SFChT and LT at the three-month visit were examined in a stepwise multiple linear regression model. All p values were 2-sided and considered statistically significant when less than 0.05.

### Clinical investigators

R. L. participated in providing and caring for the study patients; X. H critically reviewed the study proposal; M-J. G participated in the collected data.

### Ethical approval

All procedures performed in studies involving human participants were performed in accordance with the ethical standards of the Second Hospital of Dalian Medical University and the 1964 Helsinki Declaration and its later amendments or comparable ethical standards.

### Consent to participate

Informed consent was obtained from the subjects after explanation of the nature of the study.

## Results

### General characteristics

From June 2020 to October 2021, of the 46 subjects enrolled in the Ortho-K group, 42 completed all of the study visits. Among these subjects, one was disqualified because of conjunctivitis, and three subjects who missed follow-up examinations were excluded. Of the 25 subjects enrolled in the control group, a total of 20 completed the follow-up. Two subjects withdrew from the study due to other treatments (atropine), and three subjects who missed follow-up examinations were excluded. No statistically significant differences were found between the two groups at baseline (Table 1).

**Table 1:** Demographic and biometric and choroidal parameters (mean ± SD) at baseline between two groups.

Participant characteristics	Ortho-k (N = 42)	Control (N = 20)	P-values
Gender	24F/18 M	10F/10 M	0.60
Age (years)	9.45 ± 1.37	9.57 ± 1.31	0.75
SER (D)	− 2.63 ± 1.13	− 2.67 ± 1.26	0.90
IOP,(mm Hg)	15.24 ± 1.51	15.45 ± 1.67	0.62
VCD (mm)	17.21 ± 0.61	17.27 ± 0.88	0.78
AL (mm)	24.40 ± 0.64	24.34 ± 0.93	0.82
SFChT (*μm)*	282.26 ± 57.40	282.75 ± 48.38	0.97
LA-H (mm^2^)	0.28 ± 0.06	0.27 ± 0.05	0.74
LA-V (mm^2^)	0.28 ± 0.07	0.28 ± 0.05	0.79
TCA-H (mm^2^)	0.42 ± 0.09	0.42 ± 0.07	0.91
TCA-V (mm^2^)	0.43 ± 0.09	0.43 ± 0.07	0.93
CVI-H (%)	65.45 ± 2.93	64.52 ± 2.61	0.23
CVI-V (%)	65.81 ± 2.93	65.13 ± 2.07	0.36
FVs (%)	8.81 ± 0.72	8.51 ± 0.63	0.11

Subjects data presented are means ± standard deviations, except for gender. SER: spherical equivalent refractive error, IOP: intraocular pressure, VCD: vitreous chamber depth, AL: axial length, SFChT: sub-foveal choroidal thickness, LA-H: choroidal vascular luminal area on horizontal scan, LA-V: choroidal vascular luminal area on vertical scan, TCA-H: total choroidal area on horizontal scan, TCA-V: total choroidal area on vertical scan, CVI-H: choroidal vascularity index on horizontal scan, CVI-V: choroidal vascularity index on vertical scan, FVs: choriocapillaris flow voids.

P value determined by independent sample t tests, and p < 0.05 at two tails was considered to be statistically significant.

### Repeatability of then choroidal structural measurement

The ICC values of the SFChT, LA, TCA, and CVI ranged from 0.959 to 0.999 for inter-examiner repeatability and from 0.972 to 0.999 for intra-examiner repeatability in the vertical and horizontal scans (Supplementary Tables S1). Bland–Altman plot analysis indicated excellent intra- and inter-rater reliability for the horizontal scan (CVI-H) (Supplementary Fig. S3a, d), and vertical scan (CVI-V) (Supplementary Fig. S3b, e), and the SFChT(Supplementary Fig. S3c, f) was excellent. Considering the means of these choroidal parameters, the repeatability of the manual correction was good.

### Changes in the ocular biometric parameters at different time points

At the 1-month and 3-month visits, the AL and VCD were slightly increased in the Ortho-K group (*repeated-measures ANOVA* ,AL:1-month, −0.01 ± 0.05 mm, P = 0.32, 3-month, 0.03 ± 0.05 mm, P = 0.01, VCD: 1-month, 0.03 ± 0.03 mm, p < 0.001, 3-month, 0.06 ± 0.03 mm, P < 0.001, respectively), the AL and VCD were significantly increased in the control group (*repeated-measures ANOVA*, AL:1-month, 0.03 ± 0.01 mm, P < 0.001, 3-month, 0.13 ± 0.23 mm, P = 0.04, VCD: 1-month, 0.05 ± 0.03 mm, p < 0.001, 3-month, 0.15 ± 0.23 mm, P = 0.02, respectively), and the changes in the AL and CVD between the control group and Ortho-K groups were statistically significant (*one-way ANOVA analyses*, AL: 1-month, F = 17.01, p < 0.001,3-month, F = 8.73, p = 0.004, CVD: 1-month, F = 4.39, P = 0.04, 3-month, F = 6.96, P = 0.01, respectively, Table 2).

**Table 2:** Changes (mean ± SD) from baseline in ocular biometric parameters during 3 months of follow-up in two groups.

	Ortho-K group	Control group	P-values
**AL (mm)**			
Change 0ver 1 m	− 0.01 ± 0.05	0.03 ± 0.01*	< 0.001**
Change 0ver 3 m	0.03 ± 0.05*	0.13 ± 0.23*	0.004**
**VCD (mm)**			
Change 0ver 1 m	0.03 ± 0.03*	0.05 ± 0.03*	0.040**
Change 0ver 3 m	0.06 ± 0.03*	0.15 ± 0.23*	0.011**

AL: axial length, VCD: vitreous chamber depth, m: month.

** One-way ANOVA analyses, and p < 0.05 at two tails was considered to be statistically significant.

*P < 0.05 by repeated-measure ANOVA. (comparison within groups).

### Changes in the choroidal parameters at different time points

In the Ortho-K group, the SFChT, LA, TCA, and CVI on the horizontal and vertical scans were significantly increased compared to baseline at week 1, month 1, and month 3 of follow-up, and the FV% was decreased (both p < 0.001, Table 3 for specific statistical methods and actual P values). The SFChT was significantly increased by 12.61 ± 5.90 μm from baseline at the 1-week visit (p < 0.001), and the magnitude of choroidal thickening with respect to the baseline visit remained unchanged at the 1-month (mean change, 11.83 ± 6.39 μm, p = 0.333) and 3-month visits (mean change, 11.52 ± 6.29 μm, p = 0.182). The CVI was significantly increased by 2.99 ± 2.07% and 3.01 ± 2.32% on the horizontal and vertical scans respectively, from baseline at the 1-week visit (p < 0.001), and the magnitude of the increase remained stable at 1 month (mean change, 2.77 ± 2.19% on the horizontal scan, p = 0.557; 3.03 ± 2.44% on the vertical scan, p = 1.000) and 3 months of follow-up (mean change, 2.75 ± 2.23% on the horizontal scan, p = 0.959; 2.98 ± 2.52% on the vertical scan, p = 1.000). The FV% was significantly reduced by 0.89 ± 0.34% from baseline at the 1-week visit (p < 0.001), and the magnitude of the FV% reduction with respect to the baseline visit remained unchanged at the 1-month (mean change, −0.86 ± 0.36%, p = 0.543) and 3-month visits (mean change, −0.84 ± 0.33%, p = 0.138).

**Table 3:** Changes (mean ± SD) from baseline in choroidal parameters during 3 months of follow-up in the Ortho-K group.

Parameters	During Ortho-k			
	1-day	1-week	1-month	3-month
SFChT (μm)	0.38 ± 2.05	12.61 ± 5.90*	11.83 ± 6.39*	11.52 ± 6.29*
LA-H (μm^2^)	717 ± 2807	28,453 ± 17,711*	25,300 ± 15,468*	24,178 ± 15,611*
LA-V (μm^2^)	893 ± 3938	28,395 ± 14,758*	26,402 ± 14,315*	25,573 ± 15,560*
TCA-H (μm^2^)	1147 ± 3411	22,231 ± 14,815*	19,169 ± 12,779*	17,528 ± 12,751*
TCA-V (μm^2^)	391 ± 4523	22,362 ± 11,228*	20,243 ± 12,568*	19,246 ± 12,701*
CVI-H (%)	0.02 ± 0.59	2.99 ± 2.07*	2.77 ± 2.19*	2.75 ± 2.23*
CVI-V (%)	0.15 ± 0.69	3.01 ± 2.32*	3.03 ± 2.44*	2.98 ± 2.52*
FVs (%)	0.06 ± 0.25	− 0.89 ± 0.34*	− 0.86 ± 0.36*	− 0.84 ± 0.33*

SFChT: sub-foveal choroidal thickness, LA-H: choroidal vascular luminal area on horizontal scan, LA-V: choroidal vascular luminal area on vertical scan, TCA-H: total choroidal area on horizontal scan, TCA-V: total choroidal area on vertical scan, CVI-H: choroidal vascularity index on horizontal scan, CVI-V: choroidal vascularity index on vertical scan, FVs: choriocapillaris flow voids.

*P < 0.05 by repeated-measure ANOVA.

In the control group, the SFChT, FV%, LA, TCA, and CVI on the horizontal and vertical scans did not change significantly during the previous month's follow-up (all p > 0.05). At the 3-month visit, the CVI on the horizontal and vertical scans and the FV% were not significantly changed (p > 0.05). However, the SFChT, LA and TCA on the horizontal and vertical scans decreased slightly (P < 0.05, Table 4).

**Table 4:** Changes (mean ± SD) from baseline in choroidal parameters during 3 months of follow-up in the control group.

Parameters	During spectacles			
	1-day	1-week	1-month	3-month
SFChT (μm)	0.00 ± 2.81	1.15 ± 2.93	− 1.40 ± 4.14	− 3.05 ± 4.67*
LA-H (μm^2^)	− 774 ± 3724	− 2 ± 4640	− 2839 ± 5887	− 5717 ± 8013*
LA-V (μm^2^)	− 902 ± 3963	− 927 ± 4200	− 3033 ± 4937	− 4332 ± 5267*
TCA-H (μm^2^)	− 619 ± 4691	1128 ± 5691	− 1712 ± 6466	− 4229 ± 6877*
TCA-V (μm^2^)	− 530 ± 4705	674 ± 5562	− 2015 ± 5943	− 3911 ± 5809
CVI-H (%)	− 0.03 ± 0.57	− 0.12 ± 0.54	− 0.35 ± 0.68	− 0.66 ± 1.16
CVI-V (%)	− 0.13 ± 0.35	− 0.31 ± 0.50	− 0.34 ± 0.58	− 0.39 ± 0.56
FVs (%)	− 0.02 ± 0.27	0.02 ± 0.34	− 0.02 ± 0.45	0.12 ± 0.44

SFChT: sub-foveal choroidal thickness, LA-H: choroidal vascular luminal area on horizontal scan, LA-V: choroidal vascular luminal area on vertical scan, TCA-H: total choroidal area on horizontal scan, TCA-V: total choroidal area on vertical scan, CVI-H: choroidal vascularity index on horizontal scan, CVI-V: choroidal vascularity index on vertical scan, FVs: choriocapillaris flow voids.

* P < 0.05 by repeated-measure ANOVA, or P < 0.005 by Paired sample t test.

**Figure 1:**
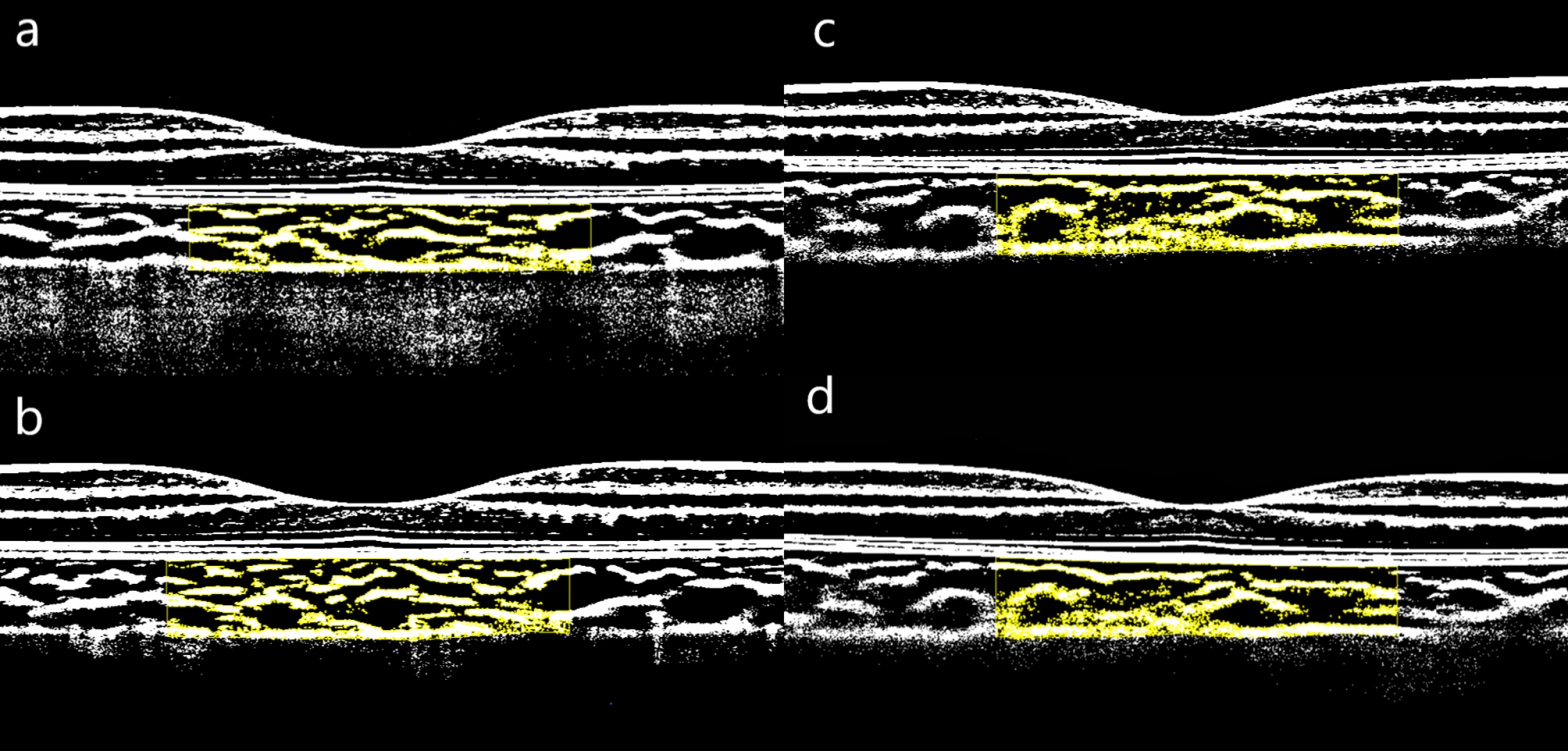
Binarized B scan images displaying the changes in CVI over time. Binarized B scan image of a patient in the Ortho-K group at baseline and week 1 of follow-up (**a**,**b**). The CVI was significantly increased by 2.51% from baseline compared to the 1-week follow-up. Binarized B-scan image of a patient in the control group at baseline and week 1 of follow-up (**c**,**d**). The CVI was reduced by 0.67%.

**Figure 2:**
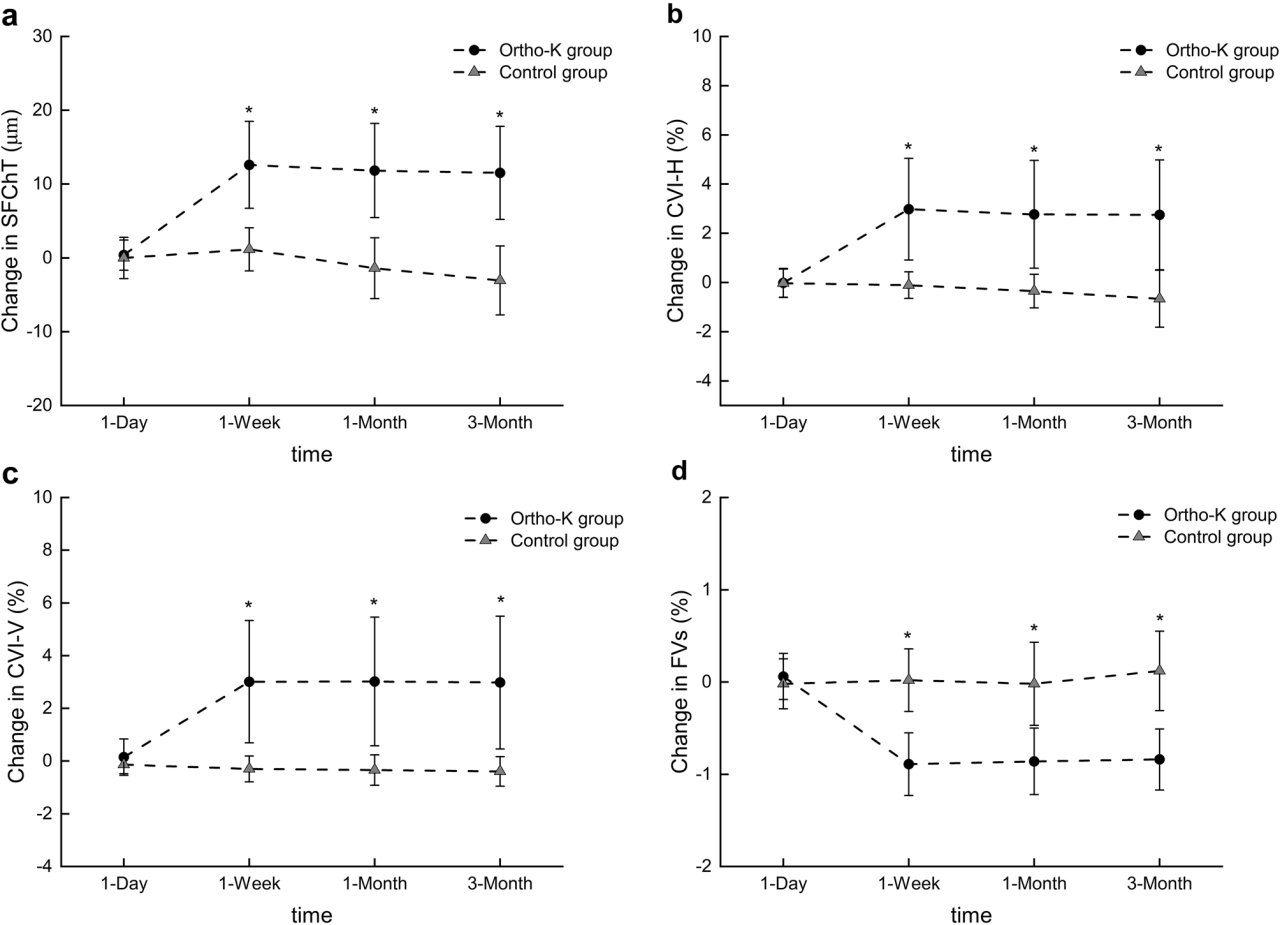
Change in the SFChT, CVI on the horizontal and vertical scans and FV% during the 3 months of follow-up between the two groups. In the Ortho-K group, the SFChT and CVI on the horizontal and vertical scans were significantly increased compared to baseline at week 1, month 1, and month 3 of follow-up, and the FV% was decreased. At the 1-week, 1-month and 3-month follow-ups, the changes in the choroidal parameters in the two groups were statistically significant The Error bars indicate the standard error (SE). *Indicated a significant difference between the two groups (p < 0.05).

Figure 1 shows binarized B scan images that displays change in CVI over time (baseline and 1-week follow-up) in a representative Ortho-K subject and a control subject. At the 1-week, 1-month and 3-month follow-ups, the changes in the choroidal parameters in the two groups were statistically significant (all p < 0.001, Fig. 2).

Correlations between the Change in the SFChT and the Change in the Choroidal Blood Flow and Ocular Biometric Parameters.

**Figure 3:**
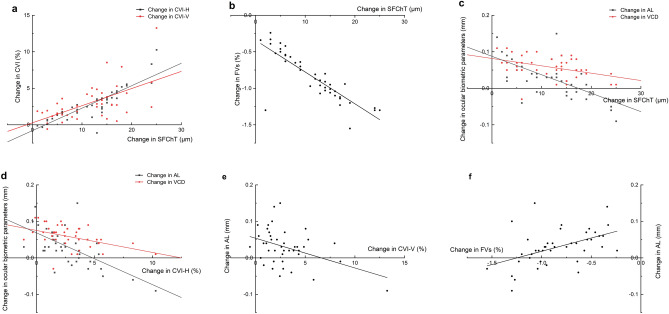
Correlation of the changes in the choroidal parameters with the changes in the ocular biometric parameters. (**a**) The change in the CVI on the horizontal and vertical scans was positively correlated with the change in SFChT; (**b**) the change in the FV% was negatively correlated with the change in SFChT; (**c**) the change in SFChT was negatively correlated with the change in the AL and VCD; (**d**) the change in CVI on the horizontal scans was negatively correlated with the change in AL and VCD; (**e**) the change in the CVI on the vertical scans was negatively correlated with the change in AL; (**f**) the change in the FV% was correlated with the change in the AL. SFChT: sub-foveal choroidal thickness, CVI-H: choroidal vascularity index on the horizontal scan, CVI-V: choroidal vascularity index on the vertical scan, FVs: choriocapillaris flow voids, AL: axial length, VCD: vitreous chamber depth.

At the 3-month visit, the increase in the CVI on the horizontal and vertical scans was significantly correlated with the increase in SFChT in the Ortho-K group (*Spearman’s correlation*, r = 0.901 and r = 0.522, all P < 0.001 respectively). A greater increase in SFChT correlated with a greater decrease in FV% (Spearman’s correlation, r = −0.827, P < 0.001). The change in SFChT was negatively correlated with the change in AL and VCD (*Spearman’s correlation*, r = −0.648, P < 0.001 and r = −0.441, P = 0.003 respectively). The change in CVI on the horizontal and vertical scans was negatively correlated with the change in AL (*Spearman’s correlation*, r = −0.580, p < 0.001 and r = −0.317, p = 0.041, respectively). The change in the CVI on the horizontal was negatively correlated with the change in the VCD (*Spearman’s correlation*, r = −0.489, P = 0.001). The change in the FV% was correlated with the change in the AL (*Spearman’s correlation*, r = −0.509, P = 0.001). (Fig. 3) There was no significant correlation between the change in SFChT and the SFChT, SE, AL, or VCD at baseline (all p > 0.05). Furthermore, the change in the CVI on the horizontal and vertical scans was not significantly associated with the baseline SFChT, SE, AL, or VCD (all p > 0.05).

## Discussion

The mechanism by which Ortho-K lenses control the progression of myopia is incompletely understood. In this study, we conducted a comprehensive analysis of short-term SFChT and ChBF in patients with low and moderate myopia treated with Ortho-K lenses and compared these subjects with those wearing single-vision distance spectacles. The CVI on the horizontal and vertical scans and the SFChT were significantly increased, while the FV% was significantly decreased after wearing Ortho-K lenses. The change in the CVI on the horizontal and vertical scans was positively correlated with that of the SFChT, consistent with previous animal studies suggesting that the bidirectional changes in ChT could be attributable to changes in ChBF^14,37,38^. Because the methods used to measure the CVI had limited lateral resolution and could not be used to identify the smaller vessels in the choroidal capillary layer^39,40^, we used en face analysis in OCTA, with the small dark areas representing the intercapillary space and highly similar to those seen on morphological and histological images^29,30^. We found that the FV% decreased significantly with the ChT in patients wearing Ortho-K lenses.

In recent years, it has been found that the choroid contains secretory cells, which might be involved in regulating vascularization and scleral growth, and the ChT can change dramatically over a short period of time to regulate the position of the retina, bringing the photoreceptors towards the plane of focus^41^. Thickening of the choroid could play an important role in the homeostatic control of eye growth and, consequently, in the aetiology of myopia. Subsequent studies have indeed confirmed this conjecture. Observations in animal models of myopia have suggested that choroidal thinning occurs early in the development of myopia and that choroidal thickening is accompanied by reduced eye growth^13,14,17^. In addition, a decrease in choroidal blood perfusion has been found in low, middle and high myopia groups^42,43^, and an increase in FVs has been found in high myopia groups^29,44^. Wu et al. found that, in adults with anisometropia, one eye with more severe myopia had a lower ChT and blood perfusion, and the severity of myopia was related to the degree of choroidal thinning^45^. However, in the present study, no significant correlation was found between the baseline ChT, CVI, and FVs with the SE, AL, and VCD values, which could be due to the similar refractive status and large individual differences in ChT in the enrolled patients.

Cross-sectional studies have some limitations. What are the roles of ChT and ChBF changes in the occurrence and development of myopia? More longitudinal studies are needed to explore this question. Currently, changes in ChT have been observed in people whose treatments, such as low-concentration atropine and Ortho-K, effectively control the progression of myopia^46,47^. Chiang ST et al. found that myopic defocus increased the thickness of the choroid and that atropine eliminated the thinning of the choroid caused by hyperopia defocus; the results showed that the additional effect of atropine and optical defocus existed at the choroidal level^48^. Whether human or animal, the refractive development of the eye is guided by the visual environment to which it is exposed^49,50^. Ortho-K lenses can be used to induce myopic defocus in the periphery in myopic children and might thus provide a potential mechanism for myopia control^51^. When negative and positive lenses are alternated, brief periods of positive lens wear balance out much longer periods of negative lens wear^52,53^, and even a 10-min myopic defocus causes transient choroidal thickening within one hour^26^. It is reasonable to assume that this transient thickening leads to subsequent persistent ocular growth inhibition. Consistent with this speculation, Li et al. found that, in children treated with Ortho-K lenses, changes in the SFChT occurred early in Ortho-K lens wear, and the short-term response of the SFChT was associated with long-term changes in the AL^24^. However, the causes of the changes in ChT remain unclear. Considering that the choroid is a highly vascularized structure capable of rapid changes in blood flow, we speculate that changes in ChBF are most likely responsible for the observed changes in ChT. Animal studies have shown that ChT changes are positively correlated with ChBP changes. Our study is the first longitudinal study of CVI and FVs changes, and it find that ChBF was significantly correlated with the AL and VCD. Our results showed significant changes in SFChT and ChBF at 1 week after Ortho-K wearing, which remained unchanged at 3 months. Compared with those in the control group, the SFChT and CVI in the Ortho-K lens group were significantly increased, and the FVs were significantly decreased. Combined with previous studies, our study further confirmed that the therapeutic effect of Ortho-K lenses extends to the choroidal and choroidal blood vessel levels and regulates the development of myopia.

How do the increases in ChBF and ChT affect the development of myopia? The mechanism is unclear, but there has been much speculation. On the one hand, there are neurons in the retina that respond in opposite directions to the wearing of a positive or a negative lens^54–56^. A cascade of signals that begins in the retina ends with molecular signals that regulate the growth of the sclera, which is not innervated; thus, these molecular signals either originate or pass through the choroid^57^. Therefore, Nickla and Wallman speculated that changes in ChT could affect eye development either by affecting molecular signals reaching the sclera or by mechanically changing the choroidal area, directly affecting the area of the sclera^41^. On the other hand, decreased ChBF might cause scleral hypoxia, while hypoxia plays an important role in the remodelling of scleral extracellular matrix and the development of myopia^58^. Moreover, studies have shown that the use of the vasodilator prazosin to actively increase ChBF could inhibit the progression of myopia, axial elongation and scleral hypoxia in guinea pigs^59^. Therefore, we hypothesized that, with improvement in the ChBF, scleral hypoxia could be alleviated, thus delaying the growth of myopia.

This study has some limitations. First, the follow-up time was too short to determine whether the early increase in SFChT and ChBF remained unchanged for a long period. The data (such as the AL and VCD data) representing the progression of myopia in the short term did not change significantly; thus, longer follow-up is needed to explore the relationship between short-term changes in the SFChT and ChBF and long-term changes in ocular biometric parameters. Second, the ocular biometric parameters measured after wearing Ortho-K lenses are not accurate, and a sufficiently long wash-out period is required to determine the changes in ocular biometric parameters after wearing Ortho-K lenses. In addition, to reduce the workload, the final image processing and analysis were performed by a single examiner. Although we selected a portion of the images and had them measured twice by two examiners, and the agreement between and within examiners was good according to the ICCs and repeatability coefficient assessment. There could still have been some potential unconscious bias affecting the measurement.

## Conclusions

In conclusion, our longitudinal study showed choroidal thickening, increased CVI and decreased FVs in the early stage in patients with low and moderate myopia who wore Ortho-K lenses. A series of choroidal changes remained unchanged over 3 months and were statistically significant compared to those in the control group wearing spectacles alone. Considered together with the positive effect of the Ortho-K lenses on myopia control in children observed in previous studies, as well as the changes in choroid thickness and blood perfusion found in this study after wearing orthokeratology lenses, we speculate that the choroid receives part of the myopia control effect induced by orthokeratology. However, more studies are needed to verify the mechanism by which ChT and choroidal blood perfusion affect the development of myopia.

## Supplementary Information

Supplementary Information 1.

Supplementary Information 2.

Supplementary Information 3.

Supplementary Information 4.

## Data Availability

The raw data are stored in FigShare, a public repository, with the following access link to https://figshare.com/s/13caa74340165798b446.
