# Supplementary figures and images for: Short-term effect of orthokeratology lens wear on choroidal blood flow in children with low and moderate myopia
Source: Sci Rep. 2022 Oct 21;12:17653. doi: 10.1038/s41598-022-21594-6 (PMC9586976; doi:10.1038/s41598-022-21594-6)

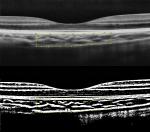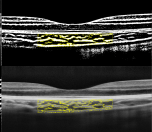

Supplement: Supplementary file 1 — Supplementary Information 1. [file 41598_2022_21594_MOESM1_ESM.pdf]

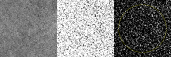

Supplement: Supplementary file 2 — Supplementary Information 2. [file 41598_2022_21594_MOESM2_ESM.pdf]
