# Supplementary material for: Short-term effect of orthokeratology lens wear on choroidal blood flow in children with low and moderate myopia
Source: Sci Rep. 2022 Oct 21;12:17653. doi: 10.1038/s41598-022-21594-6 (PMC9586976; doi:10.1038/s41598-022-21594-6)
Supplement: Supplementary file 3 — Supplementary Information 3. [file 41598_2022_21594_MOESM3_ESM.pdf]

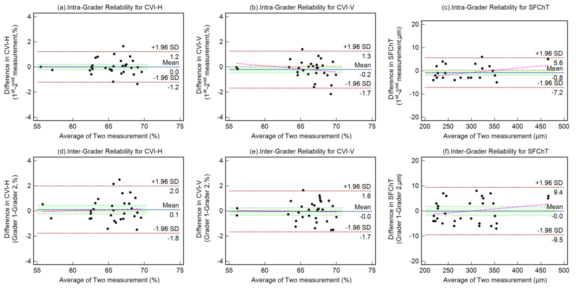
(a).Intra-Grader Reliability for CVI-H
(b).Intra-Grader Reliability for CVI-V
(c).Intra-Grader Reliability for SFChT
(d).Inter-Grader Reliability for CVI-H
(e).Inter-Grader Reliability for CVI-V
(f).Inter-Grader Reliability for SFChT
Difference in CVI-H (1st-2nd measurement,%)
Difference in CVI-V (1st-2nd measurement,%)
Difference in SFChT (1st-2nd measurement,μm)
Difference in CVI-H (Grader 1-Grader 2,%)
Difference in CVI-V (Grader 1-Grader 2,%)
Difference in SFChT (Grader 1-Grader 2,μm)
Average of Two measurement (%)
Average of Two measurement (μm)
+1.96 SD
Mean
-1.96 SD
1.2
0.0
-1.2
1.3
-0.2
-1.7
5.6
-0.8
-7.2
2.0
0.1
-1.8
1.6
-0.0
-1.7
9.4
-0.0
-9.5
