# Supplementary material for: Short-term effect of orthokeratology lens wear on choroidal blood flow in children with low and moderate myopia
Source: Sci Rep. 2022 Oct 21;12:17653. doi: 10.1038/s41598-022-21594-6 (PMC9586976; doi:10.1038/s41598-022-21594-6)
Supplement: Supplementary file 4 — Supplementary Information 4. [file 41598_2022_21594_MOESM4_ESM.pdf]

Short-term effect of orthokeratology lens wear on choroidal blood flow in children with low and moderate myopia

Qing Zhu[1], Qi Zhao[2,*]

[1, 2*] Department of Ophthalmology, the Second Hospital of Dalian Medical University, Dalian, 116027, China.
*Corresponding author(s). E-mail(s): zhaoqizq63@163.com;
These authors contributed equally to this work.

Supplementary material

Table S1. Intraclass correlation coefficients of choroidal parameters within and between examiner.

	Inter-Examiner ICC(95%CI)	Intra-Examiners ICC(95%CI)
SFChT	0.997 (0.995 to 0.999)	0.998 (0.997 to 0.999)
LA-H	0.998 (0.996 to 0.999)	0.999 (0.998 to 0.999)
LA-V	0.998 (0.995 to 0.999)	0.998 (0.996 to 0.999)
TCA-H	0.999 (0.998 to 1.000)	0.999 (0.999 to 1.000)
TCA-V	0.999 (0.997 to 0.999)	0.999 (0.997 to 0.999)
CVI-H	0.959 (0.917 to o.980)	0.983 (0.965 to 0.992)
CVI-V	0.969 (0.936 to 0.985)	0.972 (0.942 to 0.987)

SFChT: sub-foveal choroidal thickness, LA-H: choroidal vascular luminal area on horizontal scan, LA-V: choroidal vascular luminal area on vertical scan, TCA-H: total choroidal area on horizontal scan, TCA-V: total choroidal area on vertical scan, CVI-H: choroidal vascularity index on horizontal scan, CVI-V: choroidal vascularity index on vertical scan, FVs: choriocapillaris flow voids

Supplementary Fig. S1 OCT images of the choroid converted to binarization images. Segmentation block (1.5mm) of the subfoveal choroidal area (top left). The image was converted to a binary image using ImageJ software. The luminal area and the stromal area can be seen (bottom left). The rectangle surrounded by a red line was excised, and the dark areas were traced by the Niblack method (top right). The binarized image and the margin of the traced area are merged, which shows that the traced areas coincide with the dark areas of the choroidal areas of the OCT image (bottom right).

Supplementary Fig. S2 Illustration of the en face choriocapillaris image analysis. En face choriocapillaris image of the 3 × 3 mm OCTA scan region (left). Automatic local thresholding was performed with the Phansalkar method using a radius of 15 pixels to produces a thresholded image (middle). Inverted binarized images of FVs with the 2.5-mm-diameter circular region (right).

Supplementary Fig. S3 Bland-Altman plots of the SFChT and CVI. Bland-Altman plot analysis of intra- and interrater reliability for the CVI-H(1a, d), CVI-V (b, e), and SFChT(c, f). SFChT: sub-foveal choroidal thickness, CVI: choroidal vascularity index, CVI-H: choroidal vascularity index on the horizontal scan, CVI-V: choroidal vascularity index on the vertical scan.
